# *In Vitro* Biocompatibility of Si Alloyed Multi-Principal Element Carbide Coatings

**DOI:** 10.1371/journal.pone.0161151

**Published:** 2016-08-29

**Authors:** Alina Vladescu, Irina Titorencu, Yuri Dekhtyar, Victor Jinga, Vasile Pruna, Mihai Balaceanu, Mihaela Dinu, Iulian Pana, Viktorija Vendina, Mariana Braic

**Affiliations:** 1 National Institute for Optoelectronics, Magurele-Bucharest, Romania; 2 Institute of Cellular Biology and Pathology "Nicolae Simionescu" of the Romanian Academy, Bucharest, Romania; 3 Riga Technical University, 1Kalkustr, Rīga, Latvia; 4 Faculty of Physics, Bucharest University, Magurele-Bucharest, Romania; VIT University, INDIA

## Abstract

In the current study, we have examined the possibility to improve the biocompatibility of the (TiZrNbTaHf)C through replacement of either Ti or Ta by Si. The coatings were deposited on Si and 316L stainless steel substrates by magnetron sputtering in an Ar+CH_4_ mixed atmosphere and were examined for elemental composition, chemical bonds, surface topography, surface electrical charge and biocompatible characteristics. The net surface charge was evaluated at nano and macroscopic scale by measuring the electrical potential and work function, respectively. The biocompatible tests comprised determination of cell viability and cell attachment to the coated surface. The deposited coatings had C/(metal+Si) ratios close to unity, while a mixture of metallic carbide, free-carbon and oxidized species formed on the film surface. The coatings’ surfaces were smooth and no influence of surface roughness on electrical charge or biocompatibility was found. The biocompatible characteristics correlated well with the electrical potential/work function, suggesting a significant role of surface charge in improving biocompatibility, particularly cell attachment to coating's surface. Replacement of either Ti or Ta by Si in the (TiZrNbTaHf)C coating led to an enhanced surface electrical charge, as well as to superior biocompatible properties, with best results for the (TiZrNbSiHf)C coating.

## Introduction

The superior biocompatibility of metallic alloys containing biocompatible metals was proved to derive from the high corrosion resistance and biocompatibility of the oxides formed at their surfaces, the oxidation process being intensified in body fluids [1]. However, health problems related to metallosis (release of metallic ions from the alloys in the surrounding tissue, resulting in adverse physiological effects that lead to implant failure) have been reported [2]. The value of bone-fixation devices and implants is over 44% of the overall biomedical devices market. Because of this the use of coatings to enhance the wear and corrosion resistance and biocompatible characteristics of the metallic implants has been the focus of intensive research work [3]. Up to now, various types of hard coatings have been proposed, mostly consisting of the “classical” binary or ternary transition metal compounds such as nitrides (TiN, ZrN, NbN, TiAlN, TiHfN, TiSiN) [4–6], carbides (TiC, ZrC, NbC, TaC) [7–11] or carbonitrides (TiCN, ZrCN, TiAlN) [5,12–15] that exhibit superior biocompatibility as compared to pure metals. Recently, a new class of multicomponent coatings, with already confirmed biocompatible qualities [16], has been developed. These coatings, based on the concept of high entropy alloys (HEA) [17] and commonly known as multi-principal element (MPE) coatings, were produced either as metallic or as MPE compound (nitride or carbide) films. These coatings contain at least five principal elements in almost equiatomic percentage and form either simple crystalline solid solutions or amorphous structures [17–19]. The properties of the coatings can be engineered by the proper choice of the constituents. Different valuable characteristics such as high hardness, stiffness and toughness, high thermal stability, hydrophobicity, super-elasticity, superior wear, corrosion and oxidation resistance, reported for MPE coatings, are determined by their high mixing entropy, reduced diffusion kinetics, severe lattice distortion, and “cocktail effect” [19][20]. Various MPE nitride coatings have been prepared (e.g. (TiHfZrVNb)N [21,22][20,21], (ZrTaNbTiW)N [23], (TiVCrZrHf)N [24,25]), while studies on MPE carbide or carbonitride coatings are limited (e.g. (CuSiTiYZr)C [26], (CrCuNbTiY)C [27] (AlCrTaTiZr)N_x_C_y_ [28]).

In our previous papers, we investigated the mechanical and tribological characteristics, as well as the corrosion resistance and biocompatibility of a MPE carbide coating, namely (TiNbZrTaHf)C, which proved to be a suitable protective coating for biomedical applications [16,29]. The goal of the present study was to examine the possibility to further improve the biocompatibility of this coating through substitution of one metallic constituent (either Ti or Ta) by Si. As reported earlier, Si addition to binary or ternary carbide or nitride coatings improves their mechanical, anticorrosive and tribological properties [30,31]. The biocompatibility of Si and SiC is also well documented in the literature, being demonstrated that the presence of Si in different biomaterials determines the proliferation and differentiation of human osteoblast-like cell and accelerates the osseointegration of metallic implants [32,33]. Since cell integration with the implant surface is influenced by surfaces roughness and electrical charge [34], the possible correlation between coatings biocompatibility and these factors was also explored. Considering the role of electrostatic interactions in many biological events, it should be mentioned that charged surfaces have been proposed as being conductive to tissue integration [35,36]. In orthopaedic and dental applications, the surface-charge of the implant plays an important role in determining a good adhesion of bone cells to implant and also bone mineralization at the bone-implant interface [37–39]. The surface charge was characterized at nano- and macroscopic scale by the electrical potential and the work function, respectively. In addition, elemental compositions, chemical bonds and roughness of the deposited coatings were analysed. A special attention was devoted to the in vitro biological investigation using the osteosarcoma cells, in order to reveal the effect of Ti or Ta replacement by Si on the coatings' biocompatibility.

## Material and Methods

### Preparation of coatings

The coatings were prepared by magnetron sputtering using an ATC ORION unit (AJA Int.) equipped with 5 cathodes (2" diameter) [40]. The targets were made of pure Ti, Zr, Nb, Hf, Ta or Si (99.99% purity, from Kurt Lesker Comp.).

All coatings were deposited simultaneously on two types of substrates: Si (111) square pieces (l = 20 mm), cut from wafers (Si-Mat Silicon Materials Comp.), and 316 L discs (3 mm thick, 20 mm diameter; Grant Metal SA). The 316L discs were progressively polished using different emery papers (up to 4000 grit) and then polished with 0.5 μm diamond suspension to a R_a_ roughness of 50 nm. All the substrates were ultrasonically cleaned in ethanol alcohol and flushed with dried nitrogen. Each coating was deposited in the same run on two Si and four stainless steel substrates and then on six stainless steel substrates, in order to get the necessary number of replicates for characterisation.

The deposition chamber was initially pumped down to about 2×10^−5^ Pa, while the total gas (CH_4_ +Ar) pressure was of 0.67 Pa. Prior deposition, the samples were sputter cleaned with Ar^+^ (1keV) for 15 min. The deposition parameters were as follows: power applied to cathodes ~75 W (Ti), ~70 W (Zr), ~48 W (Nb), ~220 W (Hf), ~54 W (Ta); ~75 W (Si); total gas flow rate = 10 sccm; CH_4_/(CH_4_+Ar) flow rate ratio = 0.16; substrate bias voltage = –100 V; substrate temperature during deposition = 300°C. Deposition durations (120–140 min) were chosen to produce films of ~1.5 μm thick.

### Characterization of coatings

The elemental composition was determined by energy-dispersive X-ray spectroscopy (EDS), using a Bruker Quantax 70 EDS system. For each type of coating, EDS measurements were performed on two replicates in five different areas of each one, the results being averaged (arithmetic mean) and the standard deviation (SD) was calculated.

The phase composition and the preferred orientation were analysed by X-ray diffraction (XRD) technique using a Rigaku MiniflexII diffractometer with a CuKα radiation; measurements were carried out on one replicate of each coating.

The chemical bonds in the coatings were investigated by X-ray photoelectron spectroscopy (XPS), using a VG ESCA 3MK II spectrometer using monochromatic X-rays (Al K_α_(1486.61 eV)) and a hemispherical analyzer operated with constant pass energy. Survey spectra (low resolution, 1000 eV scan) were acquired using a pass energy of 50 eV with a step of 1 eV. High resolution spectra of the chemical species core levels were acquired over a smaller range (30 eV) with a resolution of 0.59 eV. The area of analysis was 300×700 μm^2^. All measurements were carried out in the analysis chamber in ultra-high vacuum conditions (~ 10^−7^ Pa). The XPS spectra were charge corrected to the binding energy of C1s line (285.0 eV). The deconvolution of XPS lines was performed using the Spectral Data Processor v 2.3 (SDP) software using the Gaussian-Lorentzian product [41]. The surface morphology was examined by scanning electron microscopy (SEM, XL-30–ESEM TMP) using one replicate for each type of coating.

The surface roughness was measured at meso-scale on 316 L coated samples, by surface profilometry (Dektak—Bruker), on an area of (150 × 150) μm^2^. The surface roughness at nano-scale on Si coated samples was determined by an AFM Innova Bruker microscope, on an area of (3 × 3) μm^2^. The roughness measurements carried out for each type of coating were performed on two replicates in 5 different areas randomly chosen on each one, the results being averaged.

The electrical potential was determined using the Scanning Kelvin Probe technique on a Kelvin Probe atomic force instrumentation (Solver–PRO47 microscope). The Kelvin probe force microscopy method was used for measuring the contact potential difference between the investigated sample and the tip of the atomic force probe [42], thus obtaining information on the Fermi level energy. Because the coatings with different compositions were measured with the same tip, the contact potential difference indicated the specific shift of the Fermi energy for each type of coating. The measurements were done for each type of coating on three random locations on each of the three replicates, the results being averaged (arithmetic mean). Measurement uncertainty was taken to be the SD over the entire scanned area. The electron work function (φ) was taken as an index for the surface charge at macroscopic scale. The value of φ is the minimal energy required for an electron to escape from a solid. The film composition, its crystalline structure and the electrical field induced by the surface electrical charge contribute to the value of φ. To measure φ, the pre-threshold photoelectron emission detection was performed in vacuum conditions (10^−10^–10^−11^ Pa) using an ultra-violet photon emission spectrometer [43]. The spectrometer, as described in detail in Ref. [44], is composed of a vacuum system, an UHV measurement chamber and a very sensitive electron detector for electron emission measurement, the noise current of the secondary electron multiplier being of about 0.5 electron/s [45]. The system uses a deuterium LSB-210 lamp (Lot quantum design) as UV source. The required photon energy was selected by means of a MDM2 (LOMO) monochromator, with a step <0.015 eV. The specimens were irradiated by soft UV light at 3–6 eV (the range of the expected value of φ) to release the electron and the photoelectron emission current (I) was measured. The value of φ was identified as the energy of the photons when I = 0.

In vitro biocompatibility tests were conducted using human osteosarcoma cells (MG63, American Type Culture Collection). For the in vitro tests were used six replicates of each type of coating and the assay was performed duplicates. MG63 cells were plated at a density of 5×10^5^ cells/ml and cultivated in DMEM low glucose medium (Sigma) supplemented with 10% inactivated fetal bovine serum, penicillin (100 U/ml), streptomycin (72,000 U/l) and neomycin (50 U/l). Cell viability was evaluated by MTT assay and cell morphology by actin staining. MTT assay was performed for in vitro evaluation of cell metabolism level. The cultured cells were rinsed with warm PBS and incubated for 3 h with the yellow tetrazolium MTT (3-(4, 5-dimethylthiazolyl-2)-2, 5-diphenyltetrazolium bromide) salt solution prepared in phenol red–free medium without serum. This salt was reduced in metabolically active cells by the cellular mitochondrial dehydrogenase enzymes resulting in an insoluble purple formazan, which was then solubilised with 0.1N HCl in isopropanol. The formazan solution concentration is directly correlated with cellular enzymatic reduction activity and it was determined by measuring the optical density at λ = 570 nm. For fluorescence microscopy, the culture medium was removed and the cells were rinsed with warm PBS and then permeabilized and fixed with a 4% PFA and 0.1% Triton X 100 solution in PBS. Then, the cells were washed and the nonspecific binding sites were blocked with 0.1% albumin for 15 min and then incubated for an hour with FITC (fluorescein isothiocyanate) coupled phalloid, for actin cytoskeleton detection and anti-vimentin primary antibody for vimentin network detection. The cells were washed and incubated with secondary antibody goat anti-mouse IgG Alexa Fluor 568 at a 1/2000 dilution and mounted using Fluoroshield with DAPI (4',6-diamidino-2-phenylindole). The images were recorded using an Axio Observer fluorescence microscope (Carl Zeiss) equipped with MRc5 digital camera. The data resulted from the biocompatibility tests were statistically analyzed by paired Student’s t-test (α = 0.005, as significant level of confidence).

The coated 316 L discs were used for EDS, SEM, work function, electrical potential and surface profilometry measurements, as well as for biocompatibility tests. XPS and AFM measurements were carried out on coated Si (111) substrates.

## Results and Discussion

### Elemental composition and chemical bonds

The mean values of the atomic concentrations measured for each type of coating in five random locations on each of the two replicates, together with C/(metal+Si) concentration ratio, are given in Table 1. The accepted precision in EDS measurement, when no standard sample is available (as in our case), in expressed as the relative SD (RDS) and is expressed as RDS(%) = (σ^at^/C_av_)*100, where (σ^at^ represents the calculated SD (%) of 10 measurements, 5 on each of the 2 replicates, and C_av_ represents the arithmetic mean content of the constituent element. We mention that for metallic (heavy) elements, the usual SRD value is around 1%, but if no standards are used, the SRD value is about 3%, which was reduced by using long counting (acquisition) times [46,47]. The calculated RDS values are also presented in Table 1. As seen, all coatings have C/(metal+Si) ratios close to unity (1.06–1.08).

**Table 1:** Elemental composition of the coatings (EDS).

Coating	Elemental composition (at.%)								C/(metal+Si)
	Ti	Zr	Nb	Ta	Hf	Si	C	O	
(TiZrNbTaHf)C	9.2±0.2	9.9±0.2	9.5±0.2	8.4±0.2	9.4 ±0.2	-	50.2±1.5	3.4±0.1	1.08
(SiZrNbTaHf)C	-	10.3±0.2	9.6±0.2	9.2±0.2	9.9 ±0.2	7.5±0.2	50.4±1.5	3.1±0.1	1.08
(TiZrNbSiHf)C	9.4±0.2	10.5±0.2	9.7 ±0.2	-	9.6±0.2	7.8±0.2	49.8±1.5	3.2±0.1	1.06

**Fig 1:**
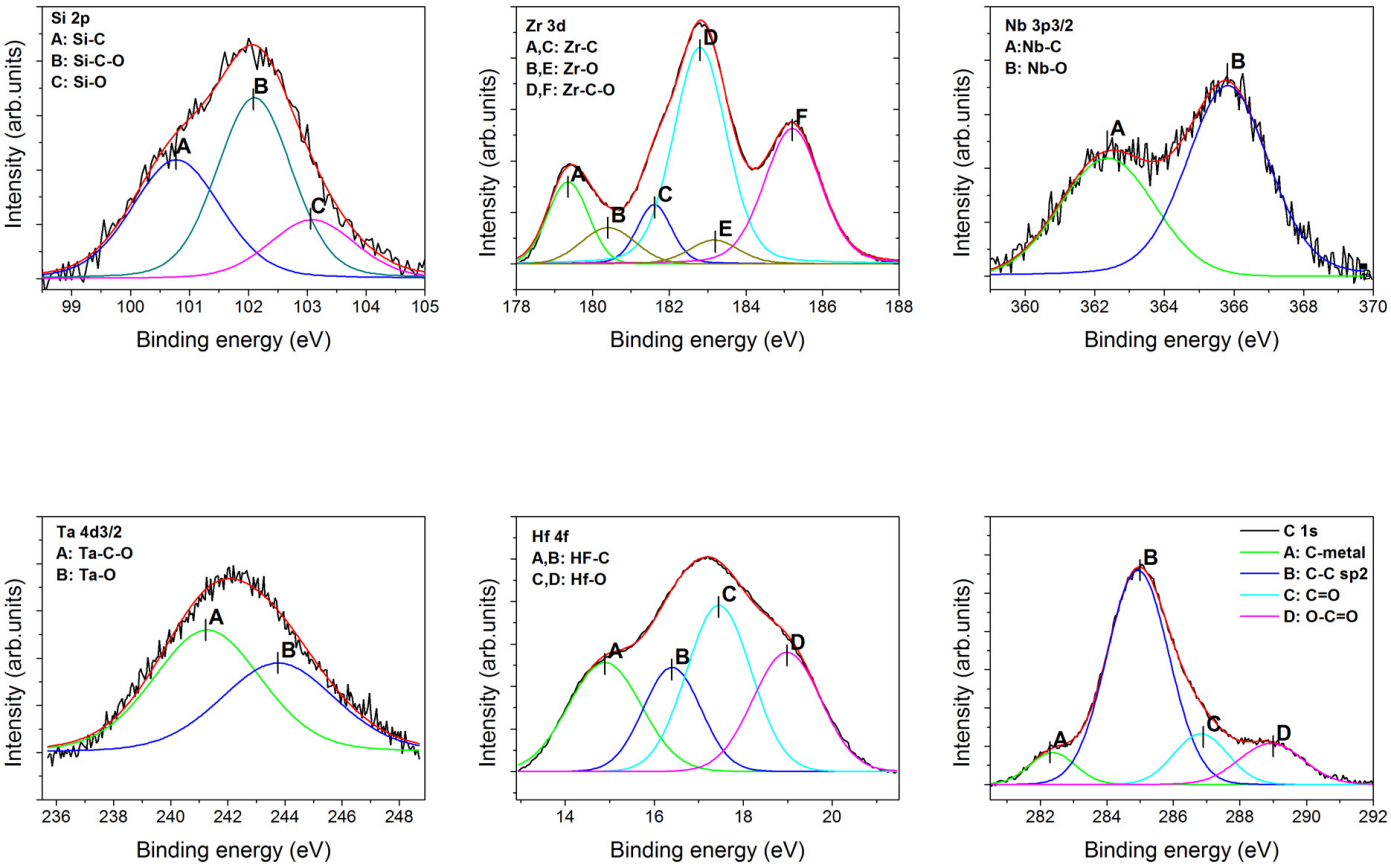
XPS Si 2p, Zr 3d, Nb3p, Ta 4d, Hf 4f and C 1s spectra of the (SiZrNbTaHf)C coating.

The XPS analysis was carried out on the surfaces of the as-deposited coatings. Fig 1 shows, as an example, the XPS Zr 3d, Nb 3p, Ta 4d, Hf 4f, Si 2p and C1s peaks of the (SiZrNbTaHf)C coating. The peak assignment was performed according to Ref. [48] as follows. The Zr 3d_5/2_ peaks at 179.4, 181.6 and 185.2 eV were associated to ZrC, ZrCO_x_ and ZrO_2_ compounds, respectively; Nb 3p_3/2_ peaks at 362.4 and 365.8 eV to NbC and Nb_2_O_5_, respectively; Ta 4d_3/2_ peaks at 241.2 and 243.9 eV to TaCO_x_ and TaO_x_, respectively; Hf 4f peaks at 14.9 and 17.5 eV to HfC and HfO_x_, respectively; Si 2p_3/2_ at 100.8, 102.1 and 103.1 eV to SiC, SiCO_x_ and SiO_2_, respectively; C1s peaks at 282.5, 285.0, 286.9 and 289.0 eV to metal carbide, C graphite and CO_x_, respectively. As resulted from the XPS analysis, the surface composition of the coatings consists of a mixture of metallic carbide, free-carbon (graphite-like) and oxides phases. The formation of surface oxides has been often reported for coatings based on transition metal compounds [49–51], being the result of the oxidation process during sample exposure in free atmosphere.

**Fig 2:**
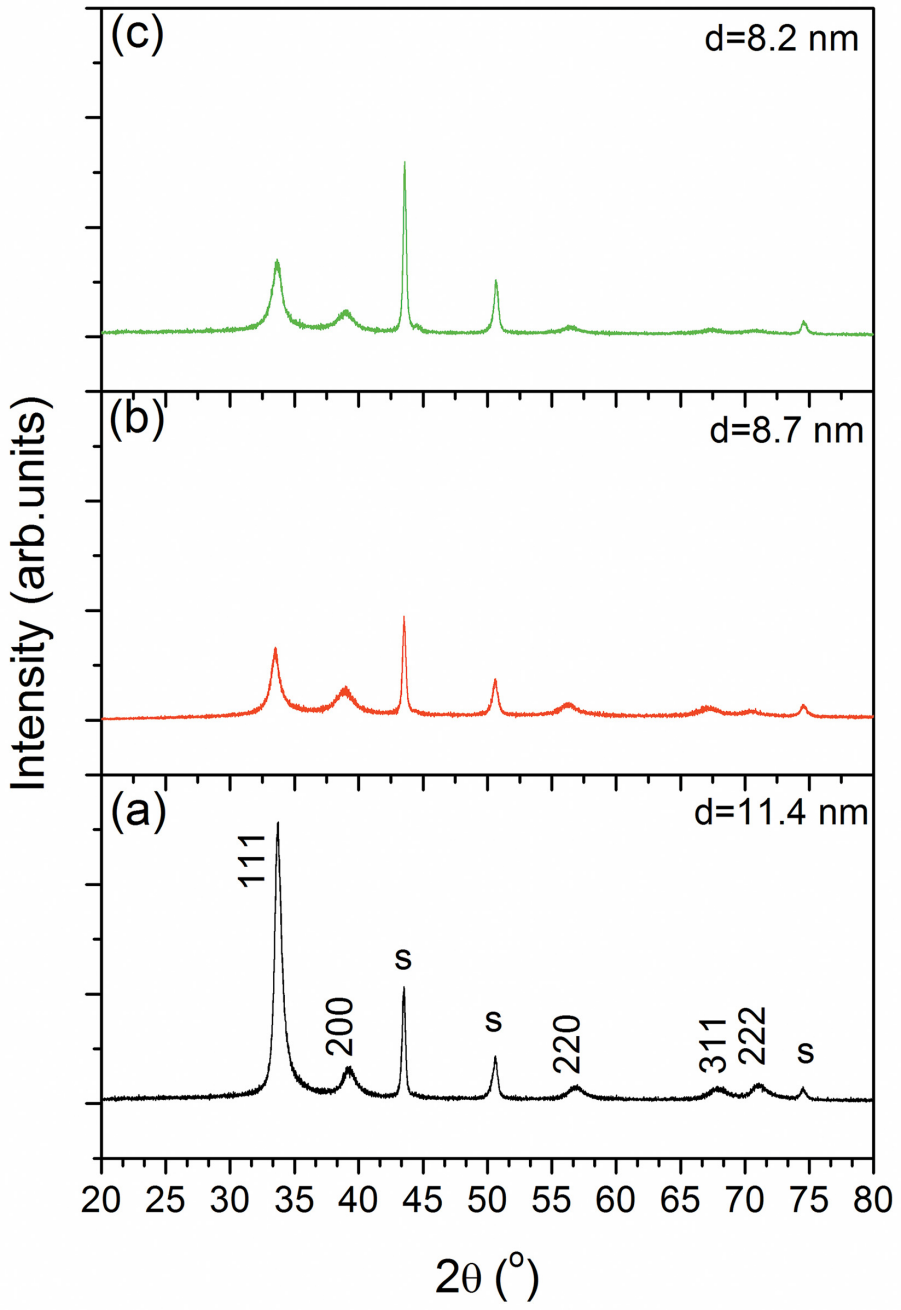
XRD diffractograms of the coating deposited on 316L substrate (S): (a) (TiZrNbTaHf)C; (b) (SiZrNbTaHf)C; (c) (TiZrNbSiHf)C; d = crystallite size.

The X-ray diffraction patterns of the coatings are presented in Fig 2. The measurements were done on one replicate of each type of coatings. The reference coating exhibited a strong (111) preferred orientation. The replacement of Ti or Ta by Si determined the decrease of the intensity of (111) peak, while the (220), (311) and (222) maxima vanished. The crystallite size was estimated from the (111) peak broadening using the Scherrer formula (d = 0.89λ/(βcosθ)). Si addition resulted in the reduction of the crystallite size, from 11.2 nm for the reference coating to 8.7 nm for (SiZrNbHfTa)C and 8.2 nm for TiZrNbSiHf)C. The observed decrease was ascribed to the increase of the amorphous phase (C = C; Si–C–O) content, as also resulted from the XPS analysis (Fig 1, C1s spectrum).

### Surface morphology

**Fig 3:**
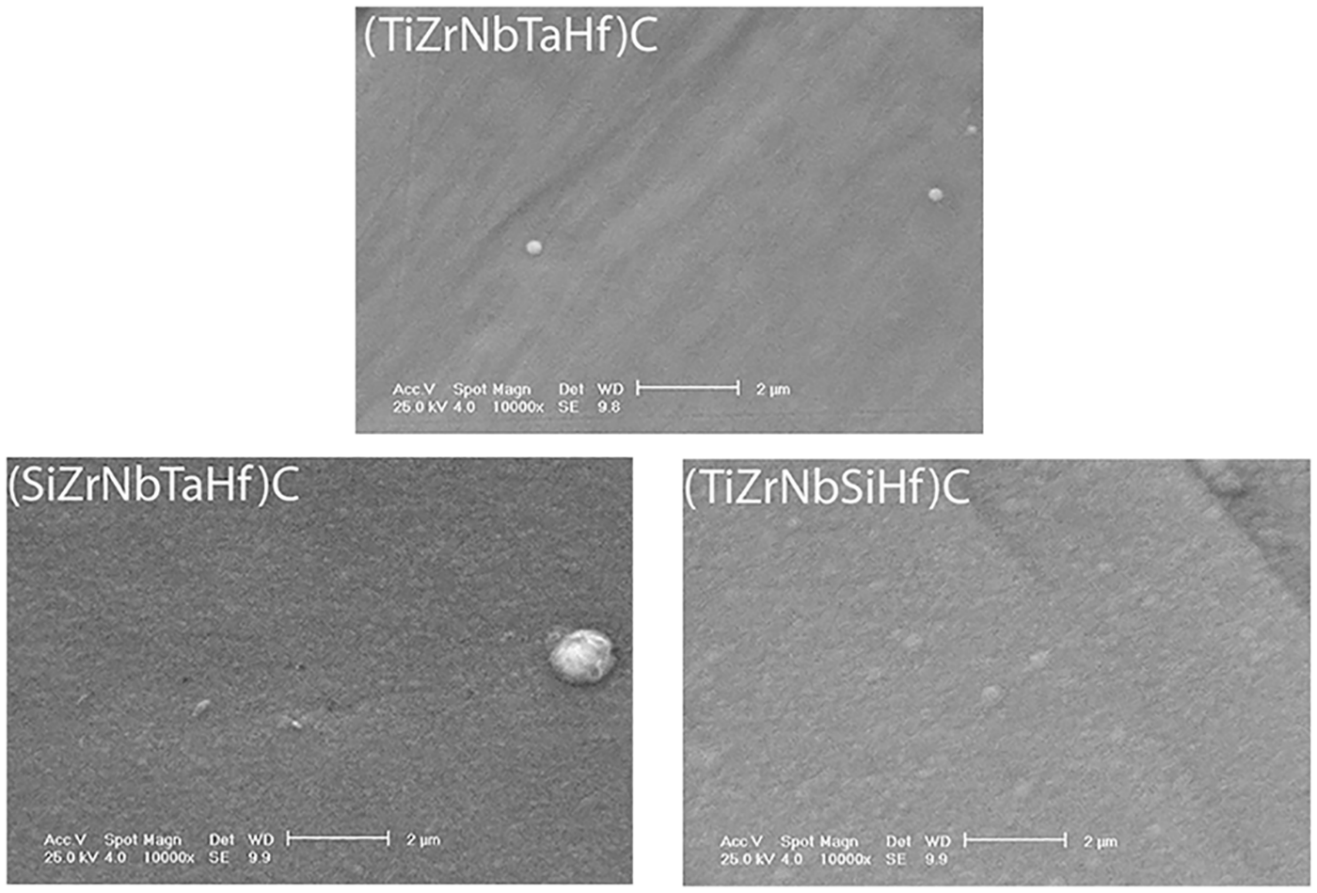
SEM surface images of the coated 316L samples. Original magnification: × 10,000.

**Fig 4:**
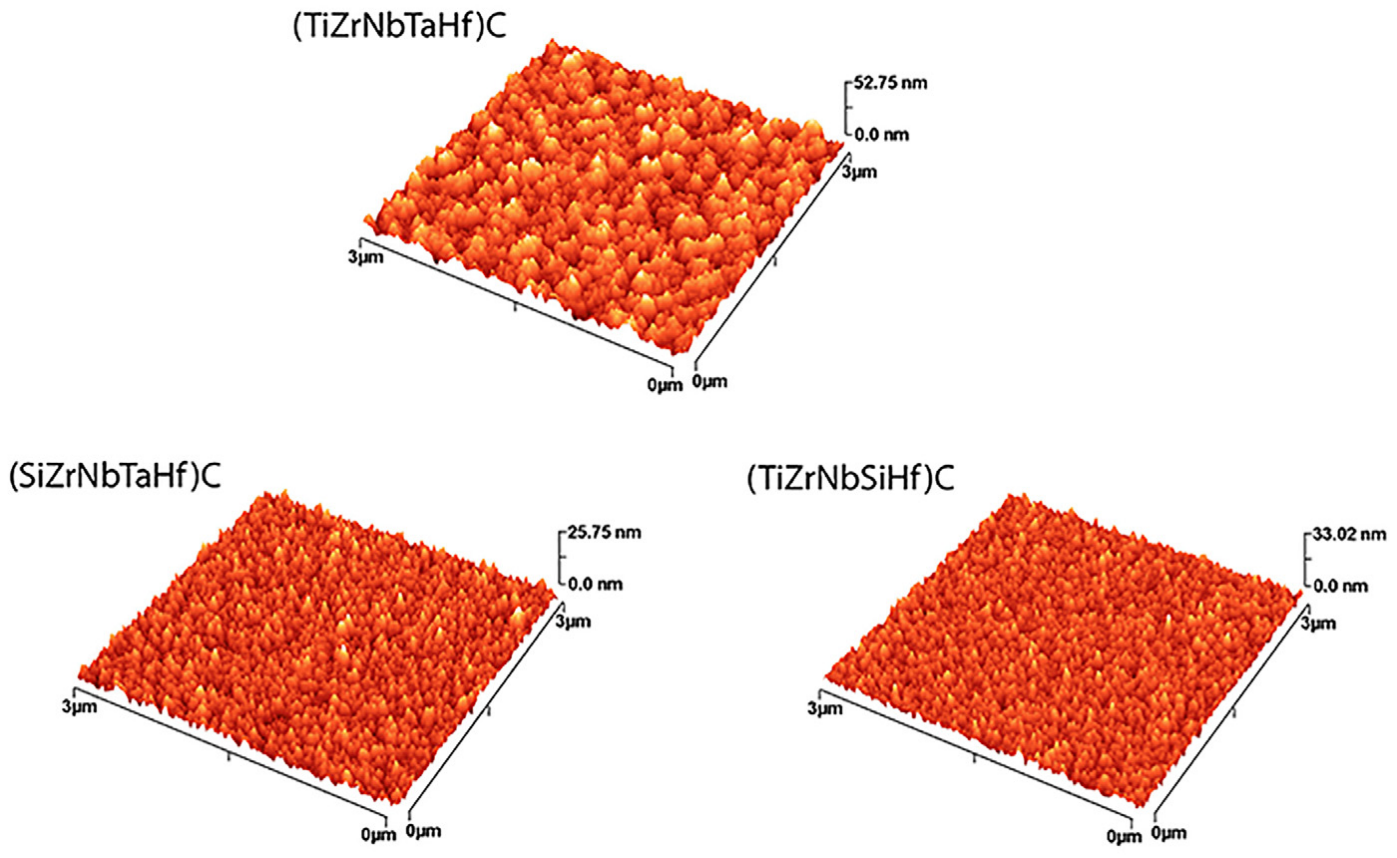
AFM surface images of the coated Si samples. Representative AFM images of the coatings: scanned area: 3 × 3 μm^2^.

**Fig 5:**
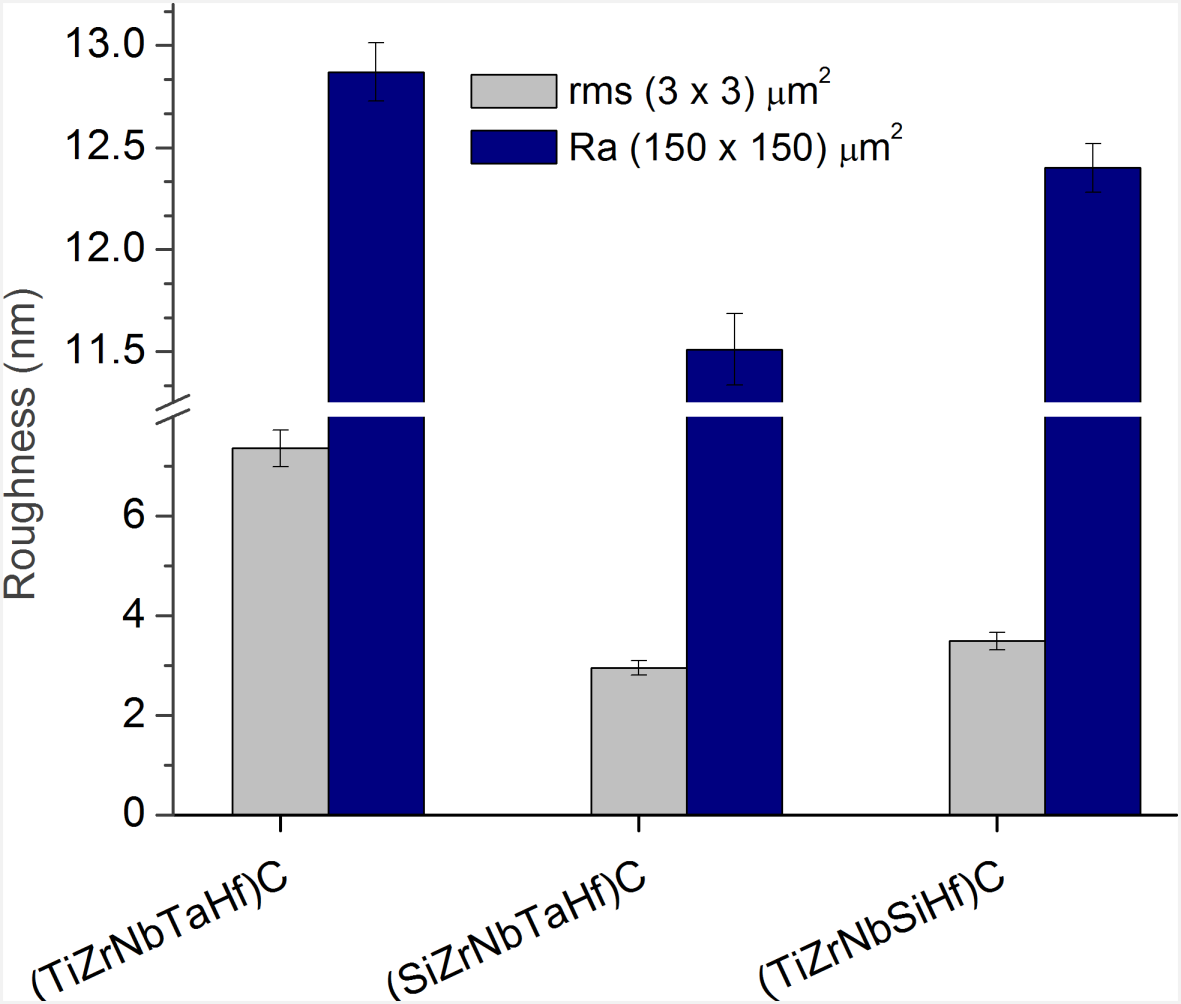
R_a_ and RMS roughness parameters of the coatings, at meso- and nano-scale. Data show the mean and SD values obtained for each type of coating on two replicates in 5 different areas. Ra roughness measurements were performed by surface profilometer (scanned area: (150 × 150) μm^2^), and the RMS roughness measurements were performed by AFM microscopy (scanned area: (3 × 3) μm^2^).

Surface morphology of the coatings deposited on 316L steel, as observed by SEM, is illustrated in Fig 3. The surfaces look smooth and dense, without cracks. The morphology of the surface at nano-scale, as measured by AFM, indicates also smooth surfaces, without pronounced hills or valleys (Fig 4). It is to note that the roughness parameters at nano- and meso-scale (RMS and R_a_, respectively) exhibited similar dependence on coating type (Fig 5).

### Electrical potential and electron work function

**Fig 6:**
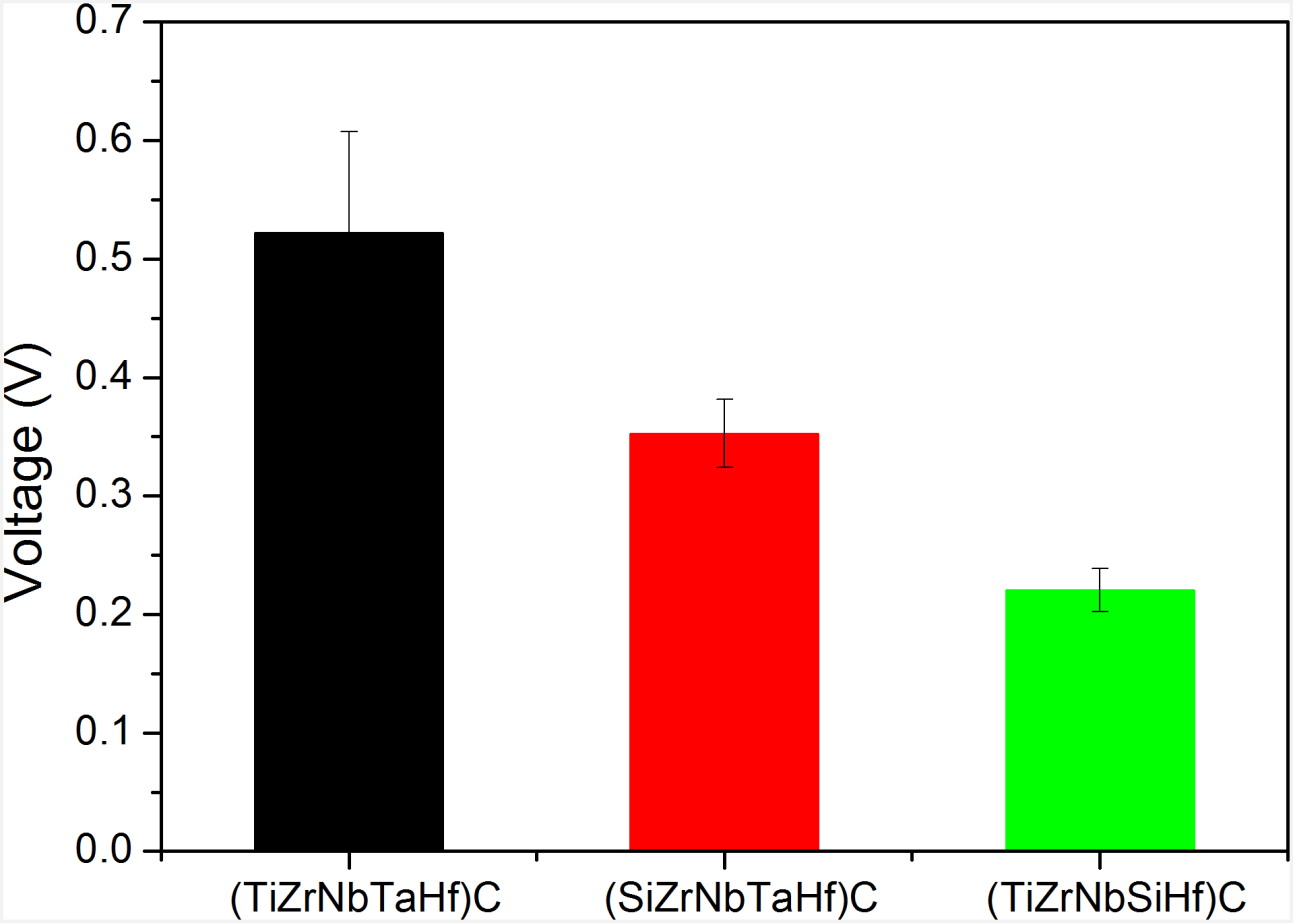
Electrical potential of the (TiZrNbTaHf)C, (SiZrNbTaHf)C and (TiZrNbSiHf)C coatings. Data show the mean and SD values obtained for each type of coating on two replicates in 5 different areas. Ra roughness measurements were performed by surface profilometer (scanned area: (150 × 150) μm^2^), and the RMS roughness measurements were performed by AFM microscopy (scanned area: (3 × 3) μm^2^).

The measurements were carried out on three replicates of each coating type, on three random locations. The surface electrical potential (V) of the investigated coatings is shown in Fig 6. As compared with the (TiZrNbTaHf)C reference, the Si containing coatings have lower electrical potentials, indicating a more negative surface charge. One may also observe that the electrical potential is not related to the surface roughness (Fig 5), probably because the roughness parameters are very low and are varying within a narrow range.

**Fig 7:**
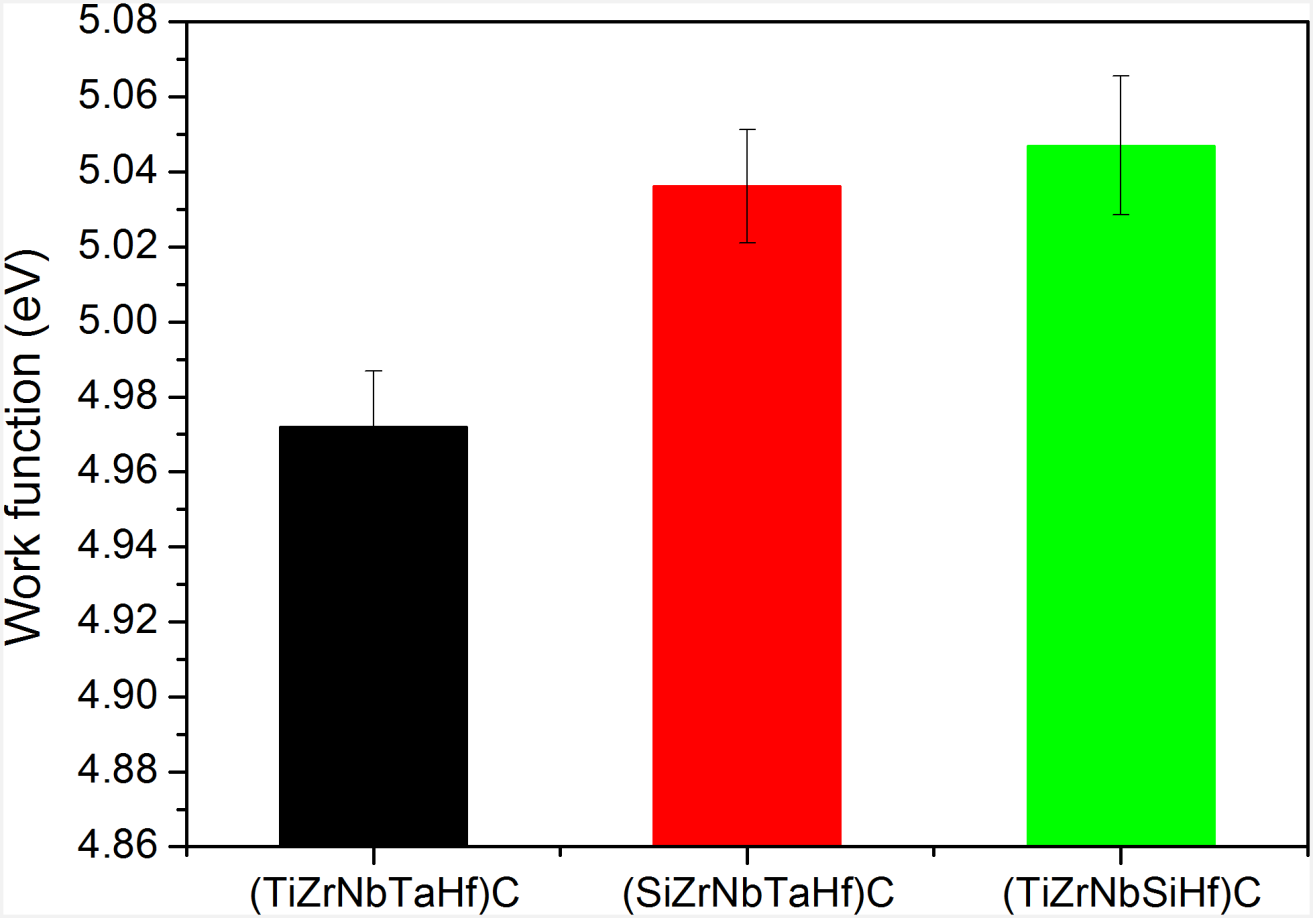
Work function of the investigated coatings. Data show the mean and SD values. For each type of coating, the measurements were done on three random locations on each of the three replicates, the results being averaged (arithmetic mean).

Electron work function of the investigated coatings is illustrated in Fig 7. The (TiZrNbSiHf)C coating exhibits the highest work function, followed by (SiZrNbTaHf)C and (TiZrNbTaHf)C. As can be seen, the changes in work function values correlate well with those of the electrical potential. This finding is in line with the results reported in Ref. [52]which showed that φ increases when the surface acquires a negative charge. Considering the measurements of the work function on polycrystalline materials, due to the random orientations of the crystallites, each area corresponding to a certain crystallite facet presents a specific value of the work function, as well documented by surface anisotropy studies [53,54]. In the current study, the work function measured at macroscopic scale represents the averaged value of all work function of the crystalline facets weighted by their area [55].

### Biocompatibility

**Fig 8:**
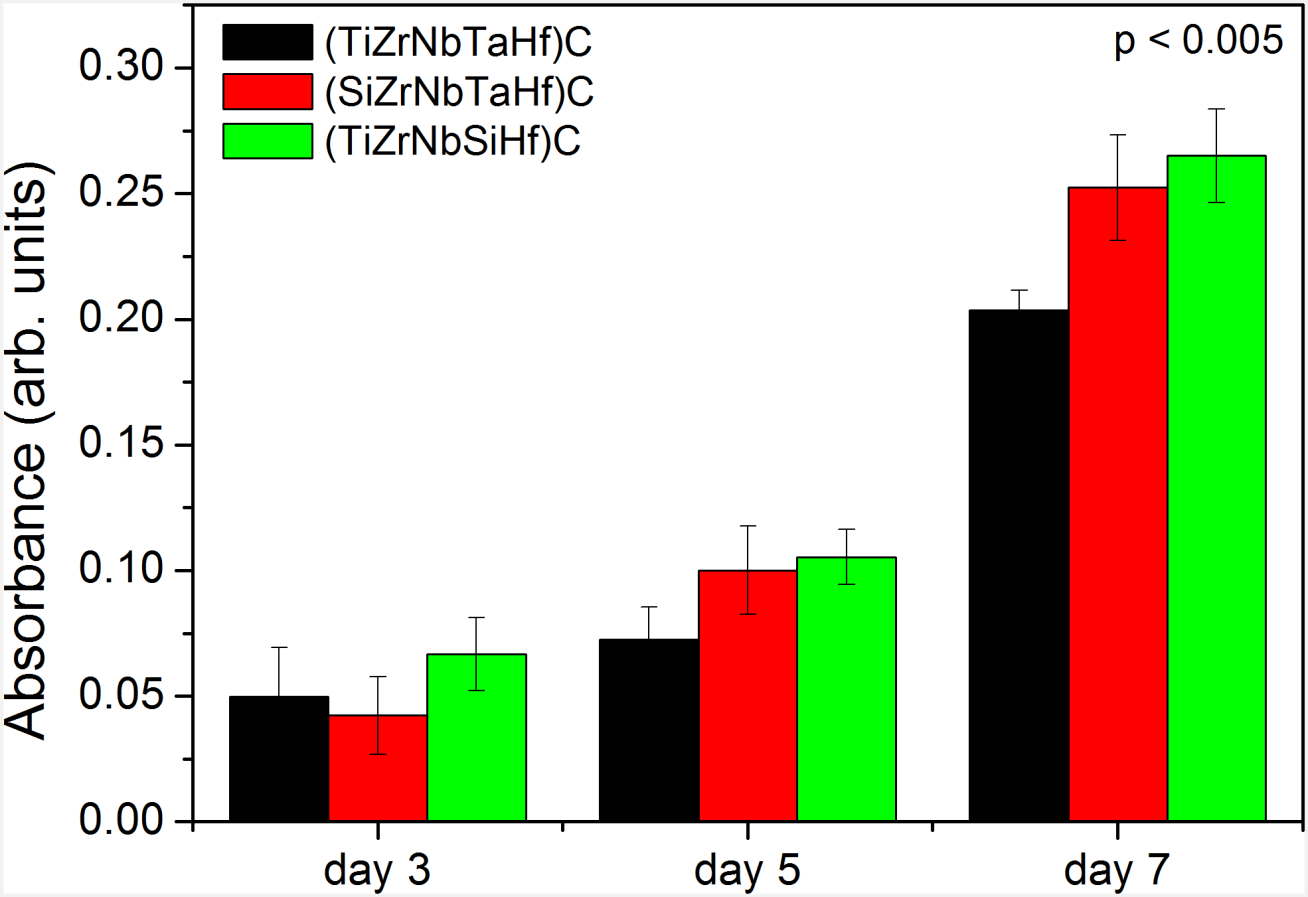
Results of MTT cell viability assay of osteosarcoma cells on the investigated coatings after 3, 5 and 7 days of culture. Data show the mean and SD values. For each type of coating, the measurements were done on 6 replicates, and the assay was performed duplicates.

Cell viability was investigated, for each coating type on six coated 316L replicates, after 3, 5 and 7 days in vitro culture using MTT test and the results are presented in Fig 8. It is clear that the cells are viable on all the coatings. After 5 and 7 days, a significant increase in the number of metabolically active cells was found for all the coatings. However, after 7 days of culture, an enhanced surface functionality of the (TiZrNbSiHf)C coating compared to the other two was observed, indicating the beneficial effect of Ta replacement by Si. Even if the differences obtained between the (SiZrNbHfTa)C and (TiZrNbSiHf)C coatings were not statistically significant, there are significant positive differences in the cell viability for the cells grown on both Si containing coatings, compared to the reference coating.

**Fig 9:**
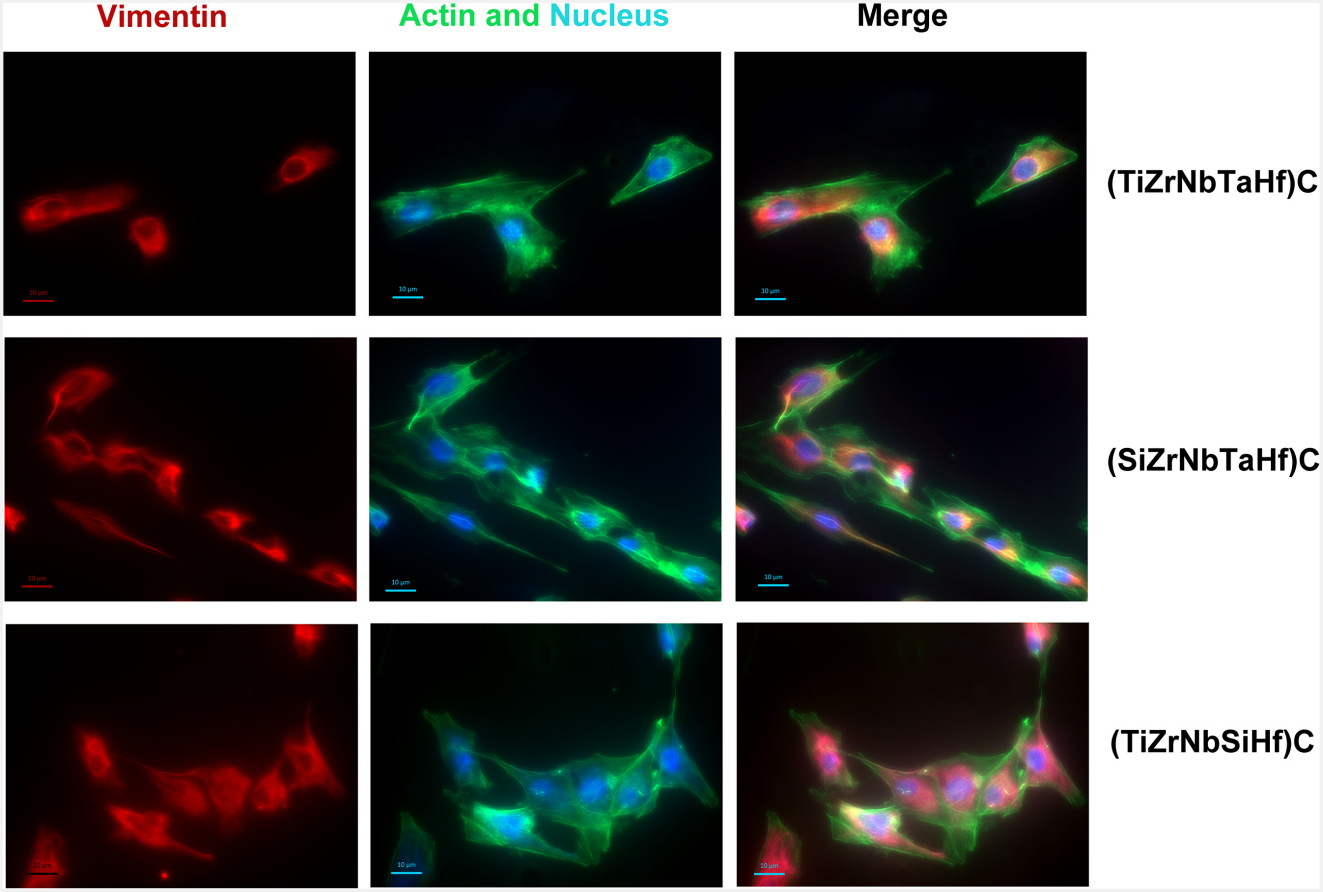
Analysis of cytoskeleton organization in MG63 cells grown after 3 days on: (TiZrNbTaHf)C, (SiZrNbTaHf)C and (TiZrNbSiHf)C. Representative fluorescent images of actin cytoskeleton and vimentin intermediate filaments were presented. Immunostaining of vimentin (purple), actin (green) are shown in separate channels. DNA was stained with DAPI (blue). Scale bar: 10 μm. Cell density was 5×10^5^ cells/ml.

The actin and vimentin filaments were labelled to observe cell morphology at 3 days after seeding the coated substrates surfaces. The actin is a main structural protein which provides information about the cells ability to adhere and spread [56], while the vimentin is responsible for maintaining cell shape and integrity [56,57], including the maintenance of the overall integrity of cytoplasm [58,59]. It is known that vimentin contributes to the construction of cytoskeleton architecture and generate cellular mechanical strength and cell integrity [59]. The immunofluorescent staining of the osteosarcoma cells on the coated substrates after 3 days (early time point) of incubation can be observed in Fig 9. Concomitant labelling of actin and vimentin was used to evaluate the cell attachment and cytoskeleton organization. The cells on all tested samples showed positive staining for actin and vimentin. The MG63 cells adhered to all surfaces and spread well, maintaining their typical spindle morphology, and b-actin was assembled into distinct filaments. Also the vimentin network displayed a predominantly perinuclear localization in cells grown on all tested coatings. Further, no differences in cell morphology were observed and there was no evidence of membrane damage, cytoplasmic vacuolation or cell death. This result shows that the cells attached and proliferated to all the tested coatings. A lower level of actin filaments and vimentin was observed for the (TiZrNbTaHf)C reference coating, indicating a limited capacity of this coating to promote cell adherence.

## Discussions

The cell attachment to an implant is a complex process that is controlled by many factors such as implant surfaces’ physico-chemical properties, crystalline structure, surface topography and roughness, characteristics of the surface oxide layer etc. The electrostatic interaction between the cells and biomaterials was the subject of numerous studies [57–62]. The experimental results demonstrated the key role played by the electrical charge of the biomaterials in cell adhesion. Superior adhesion of the osteoblast and fibroblast cells was observed for the coatings with more negatively charged surfaces [57,58,63]. It is well documented that electronegative potentials occur in non-stressed bone in areas of active growth and repair [64].

In human body, the cells, especially osteoblasts, are negatively charged [65], and it is expected to be electrostatically repelled by the negatively charged implant surface. However, Gongadze et al. demonstrated that titanium implants with low values of the surface potential promote osteoblast adhesion and the formation of the new bone [66]. The extensive review of Guo et al. [38] demonstrates that the bone cell adhesion on a biomaterial and the initial stage of bone proliferation are quite sensible to the surface charge and its polarity. Pattanayak et al. [67] reported that the negatively charged surfaces placed in a biological environment are promoting osteoblast cells-implant interaction in titanium dental implants. Krukowski et al. [36] also reported that the negatively charged surfaces promote craniofacial and intramedullary bone formation. Ohgaki et al. found that negatively charged biomaterial surfaces the cells proliferation was more intense, such as manifold layers of cells and broaden colonies of osteoblast-like cells were observed [68]. Even if the osteoblast cells are negatively charged [69], they can be attracted to negatively charged surfaces, the interaction being mediated by proteins [61,69] which are either positively charged, or which have positively charged tips, due to the presence of a quadrupolar internal charge distribution [70]. These proteins provide a substrate for the subsequent attachment of negatively charged osteoblasts. It is to note that vimentin presents a positively charged amino terminal [71]. This finding was also supported by Maroudas’ early report, which presented the dependence of cell adhesion and spreading on the surface charge of the implant [72]. Also, it was reported that many actin binding domains are rich in positive charge [73].

**Fig 10:**
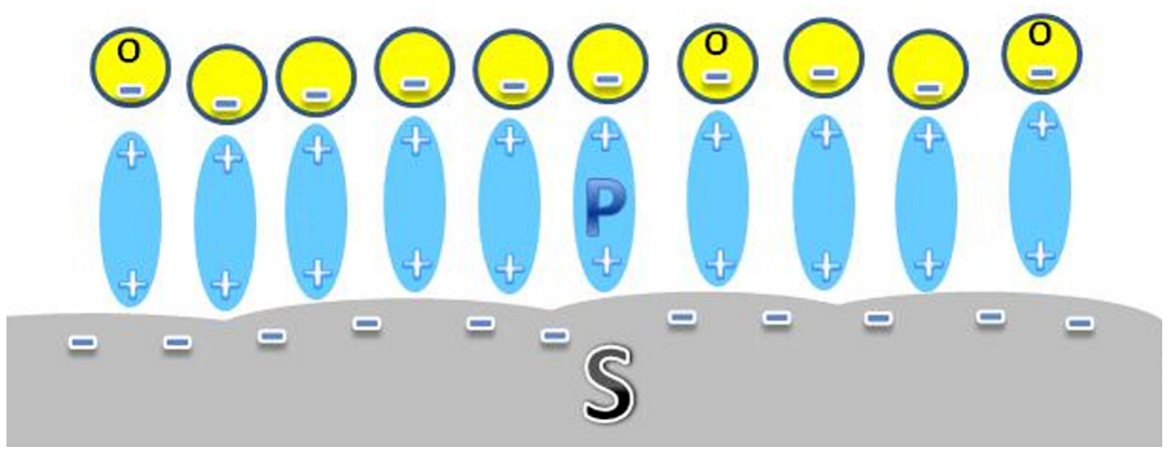
Schematic image of positive electrically charged proteins (P) attracted to a negatively charged surface (S), and the negatively charged osteoblast cells (O) attracted towards the surface.

Fig 10 presents a schematic image of the positive electrically charged proteins (P) near a negatively charged surface (S), attracting towards the surface S the negatively charged osteoblast cells (O).

However contradictory results have been also reported in the literature, as also positively charge surfaces were shown to have the same effect [34] [74].

The influence of the surface charge on cells-coating interface is certainly more complex, requiring further studies on different types of materials. According to our results, all the investigated coatings can support osteoblast cell proliferation. As compared to the reference coating, both Si containing coatings, exhibiting higher work function values and presented ~30% increase of the cell viability after 7 days of culture.

As commonly admitted, the negative surface net charge depends on elemental composition at material’s surface, topography, thickness of the surface oxide (e.g. [59]). To increase the surface charge, several approaches have been tried such as topography modification [34] or thermal and plasma treatments [75].

**Fig 11:**
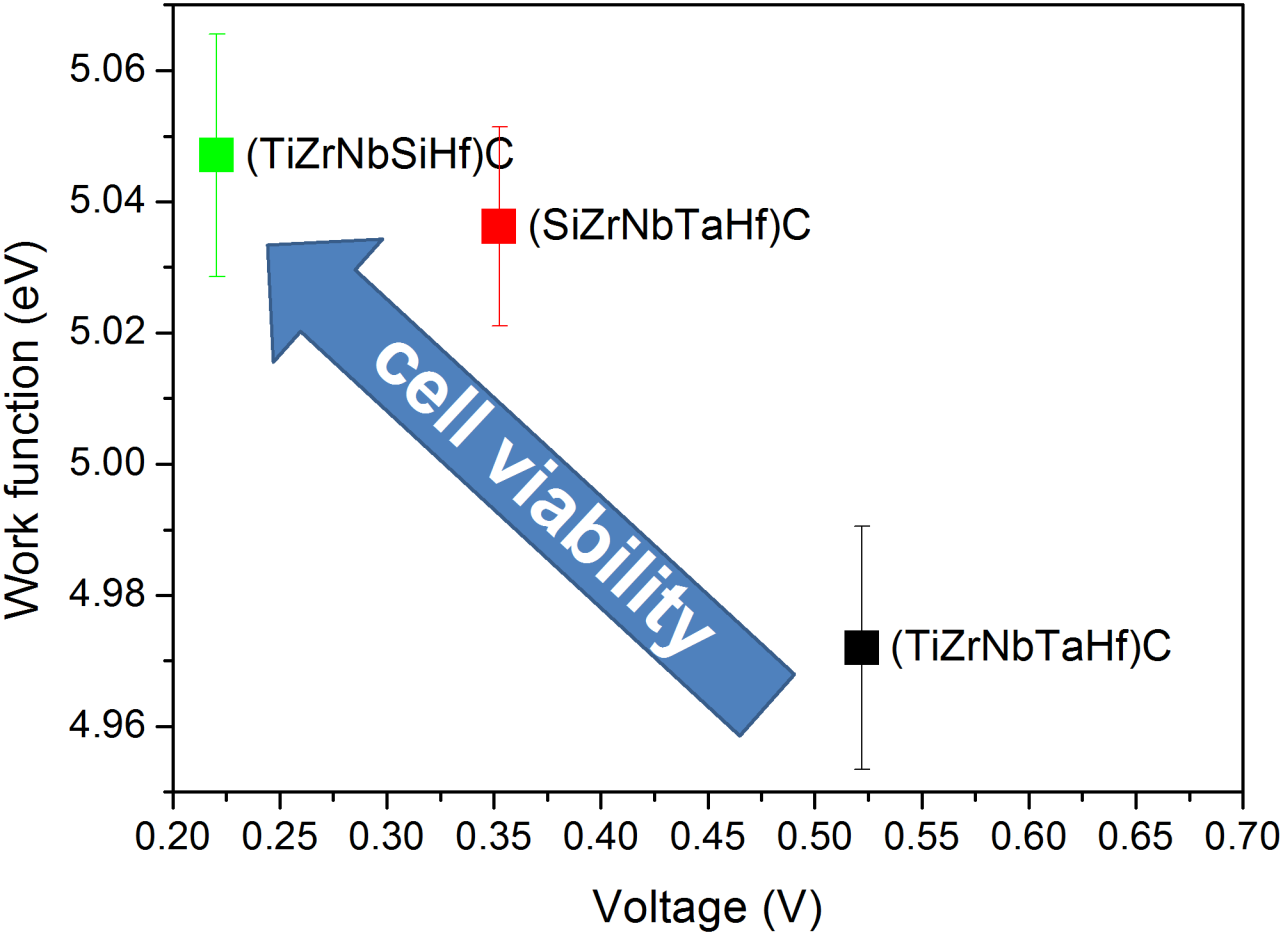
Correlation between the electrical potential, work function and cell viability of the investigated coatings.

For the coatings under investigation in this work, it was found that Ti or Ta replacement by Si led to improved cell attachment to coating surface. On the other hand, the Si containing coatings proved to have a higher surface charge compared to the (TiZrNbTaHf)C coating, as resulted from the electrical potential and work function measurements (the correlation between the electrical potential, work function and cell viability is illustrated in Fig 11). Taking into account the major role of the negatively charged surfaces in cell attachment, it is reasonable to presume that the beneficial effects induced by Si incorporation into the (TiZrNbTaHf)C coating on the cell attachment are due, at least partially, to the enhanced negative surface charge as reflected by the electrical potential decrease or work function increase. Consequently, either surface electrical potential or work function could be taken as relative predictors for evaluating a material from a biological point of view: considering a material with recognized biocompatible characteristics as reference, we could appreciate that different modifications in its composition, structure or surface properties would improve or not its biocompatibility by measuring changes in electrical potential or work function.

It is also to be noted that the effect of Si addition on coating biocompatibility is different when either Ta or Ti is removed from the reference coating. The specific mechanisms according to which one metal replacement by Si in multi-principal element carbide coatings led to an increase in the negative surface charge, may be partially ascribed to the increased Pauling electronegativity. Si (1.90) is more electronegative than Ti (1.54) or Ta (1.50), such as it was observed a net increase of the overall electronegativity of the (TiZrNbSiHf)C coating, as compared to (SiZrNbTaHf)C. It should be also mentioned that Si addition to metal carbides results in the formation of a multiphase composition consisting, beside the crystalline carbide phase, of a mixture of amorphous SiC, SiCO and SiO_x_ phases, as also resulted from our XPS measurements. Therefore, it is likely that these new phases, which have a pronounced insulator character, also contribute to the negative charge enhancement. As previously reported [59,60], in the case of Ti6Al4V alloy, the surface oxide, particularly its thickness, was found to control the net charge of the titanium alloy. It should be underlined that the measured electronegative potentials in the studied coatings were well correlated with their in vitro biocompatibility, and may be related to adequate bone growth [64] conditions.

## Conclusions

The present study explored the possibility to improve the biocompatibility of the (TiNbZrTaHf)C coating through replacement of either Ti or Ta by Si. The coatings were deposited by reactive magnetron sputtering in an Ar+CH_4_ mixed atmosphere. Considering the important role of electrostatic interactions between cells and biomaterial surface in cell attachment, the effects of surface charge as characterized by electrical potential and work function, on coatings’ biocompatibility was examined.

A significant correlation between electrical potential, work function and coating biocompatibility, as derived from osteoblasts viability and attachment to coatings surfaces, was found. Consequently, either the electrical potential or the work function are proposed as relative predictors for biocompatible characterization of the investigated coatings. Among the coatings, (TiZrNbSiHf)C, with low electrical potential and the high work function, exhibited the best biocompatible properties.
